# Anatomical and Neuromuscular Factors Associated to Non-Contact Anterior Cruciate Ligament Injury

**DOI:** 10.3390/jcm11051402

**Published:** 2022-03-03

**Authors:** Marc Dauty, Vincent Crenn, Bastien Louguet, Jérôme Grondin, Pierre Menu, Alban Fouasson-Chailloux

**Affiliations:** 1 CHU Nantes, Service de Médecine Physique et Réadaptation Locomotrice, University Hospital of Nantes, Hôpital St. Jacques, 85 rue Saint Jacques, 44093 Nantes, France; marc.dauty@chu-nantes.fr (M.D.); jerome.grondin@chu-nantes.fr (J.G.); pierre.menu@chu-nantes.fr (P.M.); 2 CHU Nantes, Service de Médecine du Sport, University Hospital of Nantes, Hôpital St. Jacques, 85 rue Saint Jacques, 44093 Nantes, France; bastien.louguet@chu-nantes.fr; 3 INSERM UMR U1229 Regenerative Medicine and Skeleton, RMeS, Nantes University, 44035 Nantes, France; 4 IRMS—Institut Régional de Médecine du Sport, Hôpital St. Jacques, 85 rue Saint Jacques, 44093 Nantes, France; 5 CHU Nantes, Clinique Chirurgicale Orthopédique et Traumatologique, Hôtel-Dieu, 44000 Nantes, France; vincent.crenn@chu-nantes.fr

**Keywords:** knee, ACL injury, sport, hamstring, strength, laxity

## Abstract

The majority of anterior cruciate ligament (ACL) injuries occur during non-contact mechanisms. Knowledge of the risk factors would be relevant to help prevent athletes’ injuries. We aimed to study risk factors associated with non-contact ACL injuries in a population of athletes after ACL reconstruction. From a cohort of 307 athletes, two populations were compared according to the non-contact or contact mechanism of ACL injury. Gender, age and body mass index (BMI) were reported. Passive knee alignment (valgus and extension), knee laxity (KT-1000 test), and isokinetic knee strength were measured on the non-injured limb. The relationship between these factors and the non-contact sport mechanism was established with models using logistic regression analysis for the population and after selection of gender and cut-offs of age, BMI and knee laxity calculated from Receiver Operating Characteristics curve area and Youden index. Age, BMI, antero-posterior laxity, isokinetic knee strength, passive knee valgus and passive knee extension were associated with non-contact ACL injury. According to the multivariate model, a non-contact ACL injury was associated with non-modifiable factors, age (OR: 1.05; *p* = 0.001), passive knee extension (OR: 1.14; *p* = 0.001), and with one modifiable factor (Hamstring strength: OR: 0.27; *p* = 0.01). For women, only passive knee valgus was reported (OR: 1.27; *p* = 0.01). Age, passive knee extension and weak Hamstring strength were associated with a non-contact ACL injury. Hamstring strengthening could be proposed to prevent ACL injury in young male athletes or in case of knee laxity.

## 1. Introduction

Every year, several hundred thousand ACL reconstructions following a sports injury are performed in the world [1,2,3]. The mechanism of ACL rupture is the most frequent non-contact injury in 70% to 75% of the cases, particularly during pivot contact sport practice [4,5]. Several risk factors have been identified to explain non-contact ACL injuries [6,7,8,9,10,11]. These risk factors are classified into two distinct categories: extrinsic or environmental (weather condition, playing surface, sport level…), and intrinsic, inherent to the individual (anatomic, neuromuscular, biomechanical, physiological, psychological and genetic factors) [4,8,10]. In this latter category, some risk factors are modifiable (e.g., body weight or muscle strength) or not (e.g., anatomical knee structure, joint laxity) because they can or cannot be controlled by the individual to reduce the ACL injury risk [10]. In the non-modifiable intrinsic risk factors, female gender and youth age (>14 or ≤20 years old) are identified as risk factors of non-contact ACL rupture [12,13,14,15,16]. Knee anatomy measured by X-ray or Magnetic Resonance Imaging, such as a decreased femoral intercondylar Notch width or an increased medial or lateral tibial plateau slopes are known as risk factors of non-contact mechanisms of ACL injury [8,17,18,19,20]. General joint laxity, passive knee extension (recurvatum) and anterior-posterior knee laxity seem to be risk factors for the occurrence of a non-contact ACL injury, especially in women [13,21,22,23,24,25].

Depending on modifiable intrinsic risk factors, an increase in Body Mass Index (BMI) seems to be a questionable risk factor for non-contact ACL rupture [9,11,16,21] Biomechanical and neuromuscular factors would be impaired [26]. Dynamic knee valgus would be poorly controlled in women during a non-contact ACL rupture, due to an increase in hip varus, knee valgus and Hamstring strength deficit (or an imbalance of Hamstring/Quadriceps ratio) [11,27,28,29,30,31,32]. However, the links between dynamic knee valgus and static knee valgus (non-modifiable factor) are poorly known [33,34,35].

Therefore, a better understanding of these risk factors seems necessary in order to avoid non-contact ACL ruptures during sport practice. These risk factors should be easily measurable to be useful in clinical practice [30]. The objective of this study was to investigate the association between intrinsic factors considered at risk (age, BMI, passive knee alignment, antero-posterior laxity and isokinetic strength knee) and the non-contact ACL injury. Gender has to be particularly taken into consideration because women athletes suffer ACL injury at a 2- to 6-fold greater rate than male [14,36,37]. Methodologically, it was hypothesized that the two knees were identical in a same athlete before injury and that patients who had a non-contact ACL injury had more frequently predisposing associated factors than patients with contact ACL injury. This particular method had been used because of the difficulty for many years in tracking down athletes who had not had knee surgery, pending the occurrence of a primary ACL rupture [38,39].

## 2. Materials and Methods

### 2.1. Population

All athletes over the age of 14 who had performed an isokinetic knee evaluation as part of the usual 6-month follow-up of an ACL surgical reconstruction were included from 2 January 2018 to 17 March 2020 (French COVID 19 confinement). Patients were excluded if they had undergone bilateral ACL reconstruction, a second ACL reconstruction of the same knee, an LCP reconstruction and/or multiple peripheral ligaments reconstruction, or a modification of the knee alignment by bone surgical correction. Patients were also excluded if they refused to participate in the study. Finally, three hundred and seven patients were included, 206 men and 101 women (age: 26 ± 9 years, weight: 71 ± 12 kg, height: 173 ± 8 cm, BMI: 23.4 ± 3.2 kg/m^2^). Thirty patients were excluded because of bilateral ACL reconstruction, 27 because of a second ACL reconstruction, 10 because of LCP reconstruction, 10 because of multiple ligaments reconstruction and 5 because of an operated knee axis modification. No patient refused to participate to the study.

### 2.2. Anthrometric Parameters

Age, weight and height were measured and the Body Mass Index (BMI) was calculated using the weight-related formula for square height [9].

### 2.3. Knee Anatomic Parameters

Knee anatomical parameters were measured on the contralateral healthy knee and on the knee with ACL reconstruction by the same observer (MD). The 7 days test–retest reliability of the clinical knee measurements was assessed in the first 30 subjects included, using the intra-class correlation coefficient. Knee alignment was measured in the frontal plane in a standing position according to knee morphotypes [4,6]. Passive knee valgus was quantified by measuring the inter-malleolar distance using a ruler to the nearest millimeter. The intra-examiner reliability of measurements was excellent (ICC: 0.97 (0.95–0.98)). Passive knee extension was evaluated in degrees in the sagittal plane in dorsal decubitus with a goniometer when passively extending the knee [6,40]. The intra-examiner reliability of measurements was excellent (ICC: 0.97 (0.94–0.98)).

Knee laxity was measured in millimetres by the same experimented physician using a KT-1000^®^ arthrometer (MEDmetric^TM^ Corp., San Diego, CA, USA) [11,22,40]. A cut-off ≥ 3 mm corresponds to the threshold of pathological laxity [22,41,42]. The intra-examiner reliability is good for 134 Newton [43].

### 2.4. Isokinetic Knee Parameters

Muscle strength was assessed using an isokinetic dynamometer CYBEX NORM^®^ (Lumex Inc., Ronkonkoma, NY, USA) according to an identical protocol for each subject. Knee quadriceps (extensors) and hamstrings (flexors) strength of the healthy knee were assessed in concentric mode at 60 and 180°/s angular velocities. The knee range of motion was limited to 100 degrees (from the full extension to 100 degrees of flexion). Gravity correction was used for all tests. Three repetitions at 60°/s and then 5 repetitions at 180°/s were performed. The relative isokinetic strength was calculated by reporting maximum peak torque to the bodyweight. The hamstring-to-quadriceps ratio (H/Q) was calculated for 60°/s angular speed. The reliability of quadriceps strength measurement (ICC between 0.95 and 0.98) and hamstrings strength measurement (ICC between 0.93 and 0.97) is excellent at 60 and 180°/s angular speed [44]. The reliability of H/Q ratio is good only for the 60°/s angular speed (ICC between 0.65 and 0.79) [44].

### 2.5. Definition of Non-Contact and Contact ACL Rupture

Non-contact ACL injury was defined by a knee twisting mechanism (the foot usually remained fixed to the ground while the leg rotated overstretching knee ligaments). that occurred without collision and without high kinetic reception (high-impact rotation landing).

Contact ACL injury was defined by a knee twisting mechanism that occurred when the subject came into contact with another subject on the knee or body or if there was a high kinetic reception as it is the case with a high-speed skiing fall, for example [45].

Non-contact or contact mechanism of ACL injury during sport practice and anthropometric parameters were reported by the orthopedic surgeon before ACL reconstruction. Anatomic and isokinetic evaluations were realized blindly by an independent physician.

### 2.6. Statistical Analysis

Two populations were identified according to the occurrence of the non-contact or contact ACL injury. The statistical analyses were performed using SPSS 23.0 software (Armonk, NY, USA, IBM Corp.). Quantitative parameters were presented as mean and standard deviation and qualitative parameters in frequency. Univariate analysis (independent Student t-test) and a χ^2^ test or Fisher’s exact test were used to compare quantitative and qualitative data of the non-contact and contact groups. The results were considered statistically significant at the 5% critical level (*p* < 0.05).

To confirm associations, 10 events per analyzed variable are recommended [46]. Since the objective was to analyze 8 potential intrinsic risk factors as gender, age, BMI, passive knee valgus, passive knee extension, anterior-posterior laxity and hamstring or quadriceps knee strength, more than 80 subjects were necessary. Due to a known incidence of 75% of ACL rupture without contact in the general population [5] and an incidence of 63.5% (195 of 307) of ACL rupture without contact found in our studied population, a minimum of 170 subjects were required at the end point to conduct the analysis at 0.05 type I error rate and at 0.10 type II error rate.

Multivariate analysis was assessed using the binary step by step ascendant logistic Wald regression (inclusion probability < 0.10 for associated risk factors). Logistic regression function was used to model the probability of non-contact ACL injury. Because of continuous quantitative parameters, the ORs were estimated from the exponential of the coefficient *B* of the logistic regression [47]. The Hosmer–Lemeshow test was used to describe if the data fitted the model well. The R-squares of Cox-Snell and Nagelkerke (% of the variance explained by the predictors) were used to know if the model was well adjusted.

Different models were shown in accordance to gender and after selection of cut-offs of variables identified by Youden index and ROC (Receiver Operating Characteristics) curve area to know how well this cut-off could distinguish the different non-contact groups [48]. The ROC curve area was interpreted as excellent (0.9–1), good (0.8–0.9), fair (0.7–0.8), poor (0.6–0.7), or failed (0.5–0.6) [49,50].

## 3. Results

Out of the 307 patients, 195 reported having had a non-contact ACL injury (63.5%) and 112 a contact injury during sports practice prior to ACL reconstruction (Table 1). A significant difference was found between the 2 groups for the following qualitative variables: age, weight, BMI, quadriceps and hamstring strength at 60 and 180°/s, passive knee valgus, passive knee extension and knee laxity, and they were included in the binary logistic regression model (Table 2). The overall accuracy or diagnosis efficiency of non-contact ACL injury was 63.5% from three parameters: age, Hamstring strength at 180°/s, and passive knee extension (Table 3). The data fitted the model well (Hosmer–Lemeshow test; *p* = 0.499), and the model was well adjusted (R-squares of Cox-Snell and Nagelkerke of 0.12 and 0.16, respectively). Only 1 case was not well classified.

The different cut-offs for our population are presented in Table 3. However, ROC curve areas are poor (0.6–0.7) for passive knee extension, passive knee valgus and age, and failed (0.5–0.6) for BMI and knee laxity (Table 4). Considering male gender, we found that passive knee extension and age (non-modifiable factors) and Hamstring strength (modifiable factor) were associated with the non-contact ACL injury. Considering female gender, only passive knee valgus was associated with non-contact ACL injury (Table 3). Only hamstring isokinetic strength was a modifiable and protective factor after selection of cut-offs of age ≤ 23.5 year, or of knee laxity ≥ 4.5 mm (Table 3).

## 4. Discussion

The interest of identifying associated factors with a non-contact ACL injury is to set up subsequently preventive strategies to decrease the incidence of this type of injury [51]. Because non-contact ACL injury occurrence is multifactorial, multivariate analysis was necessary to analyze the combination of factors to identify groups at risk of non-contact ACL injury. When the whole population had been studied, age and passive knee extension presented a significant association with the non-contact ACL injury. This result is interesting to advise an individual before practicing a sport at risk for the knees. However, no preventive intervention can be proposed because these two factors are not modifiable. On the contrary, the association with weak hamstring strength, considered as a protective factor (OR: 0.27), is very interesting because this factor can be improved by strengthening.

The comparison with prospective studies which proposed multivariate risk factor models is not easy because the same parameters have not been studied. However, the presence of a passive knee extension has often been found to be a risk factor for ACL knee injury, especially in female soccer or basketball players (OR from 3.8 to 4.7) [28,40]. However, the relationship with non-contact ACL injury is debatable for all athletes when this factor is evaluated individually (non-adjusted univariate model). Vauhnik et al. have shown no significant relationship (OR: 1.00 (0.93–1.16); *p* = 0.44) in women [21] and more recently, Amraee et al. have considered passive knee extension to be a non-associated factor after comparison with a non-injured population [6]. However, when this parameter is part of the general laxity, it is associated with non-contact ACL injury, whatever the gender (OR: 3.1 for men and 2.7 for women) [11]. From our results, this parameter was associated only 1.14 times with a non-contact knee injury. The difference of association can be explained by the fact that a small passive knee extension does not have the same meaning as a large passive knee extension. Thus, the presence of passive knee extension does not sufficiently reflect the risk, probably because the link is all the stronger as the passive knee extension is great. Determining a cut-off of the passive knee extension is therefore more specific than using this variable in a dichotomic way. From our population, this cut-off was of 4 degrees with a sensitivity of 61% and specificity of 58.9%. From a mechanical point of view, a knee hyperextension stresses the ACL by increasing the anterior tibial translation, which may occur at the end of a jump or during a running deceleration. In such cases, an ACL impingement on the intercondylar notch width can occur until the ligament rupture [23,24,31,32].

Passive knee valgus had already been studied as a risk factor of non-contact ACL injury but according to the Q angle method (angle between the anterior superior iliac spine-center of the patella-tibial tubercle). Knee valgus corresponds to an excessive Q angle [24]. With this parameter, no relationship was found with non-contact ACL injury [6,24]. The controversy could be explained by the Q angle method expressed in degrees, which is different from the present knee valgus measurement method expressed in millimeters. The association was 1.27 times in our population, but only in women. The best cut-off was 15 mm with a poor sensitivity of 41% and a good specificity of 79%. From a mechanical point of view, knee valgus is associated with a greater coxa vara with concurrent increase in tibio-femoral rotation force and dynamic anterior tibial translation, thus imposing greater stress on the ACL [52]. Dynamically, knee valgus, assessed by 3D motion analysis at landing, predicts ACL injury in women [37]. The fact that passive knee valgus was the only founding factor associated with non-contact ACL injury in women in our study may confirm a relationship between passive and dynamic valgus.

Body Mass Index was considered a risk factor but only in women athletes [11,16]. We have found a relationship with this parameter only in univariate analysis, but no association was confirmed after multivariate analysis in the whole population or only in the women population. In the same way, weight was identified an associated factor only in univariate analysis. After selection of the men population, no association was found after multivariate analysis. Evans et al. have shown a relationship but only in a military population different from our sport population [9]. Yet, we have shown that patients with non-contact ACL injury were at risk of injury in case of Body Mass Index ≥ 22.5 kg/m^2^. Passive knee valgus and passive knee extension increased this risk of 1.21 and 1.16 times, respectively. In this particular population, the Body Mass Index is the only modifiable factor.

In our study, the age of the non-contact ACL group was older (mean: 27 years old) than those of the contact ACL injury group (mean: 24 years old). This parameter is debatable because age was not considered a risk factor in prospective study [11,28,29]. Only Hagglund et al., have described a cut-off superior to 14 years old in a retrospective study of very young female soccer players, aged between 12 to 17 years old [16]. In contrast, age was not associated with non-contact ACL injury in two other populations aged 18 and 33 years old [15,21]. However, when the population under 23.5 years old was taken into consideration, passive knee extension and a poor Hamstring isokinetic strength were associated with non-contact ACL injury. Because hamstring isokinetic strength was a protective factor (OR from 0.09 to 0.88), strengthening this muscle group would be interesting for ACL injury prevention. Some authors have already described knee muscle strength as the objective neuromuscular risk factor of non-contact ACL injury with controversial results [11,28,29]. Myer et al. have shown weak hamstring isokinetic strength with relative great quadriceps isokinetic strength in women with ACL injury [29]. On the contrary, Uhorchak et al. have not found a particular knee strength risk of non-contact ACL injury [11]. However, the strength normalized to body weight was questionable in this study because this parameter was expressed in an unusual unit in % of the bodyweight and not in Nm/kg [11]. Therefore, the values were very different from ours and may explain the absence of the possibility to identify isokinetic knee strength as a risk factor. Soderman and al. have used bilateral knee strength symmetry index and hamstring-to-quadriceps ratio as strength parameters [28]. Bilateral symmetry indexes were not different between traumatic and non-traumatic injuries groups. However, the mean of the hamstring-to-quadriceps ratio of the two legs was lower in the traumatic group (OR: 0.93 (0.88–0.99); *p* = 0.02) [28]. We have not confirmed this result, maybe because we have only studied the hamstring-to-quadriceps ratio of the healthy knee and not the mean of the two knees.

Antero-posterior knee laxity is known to be associated with noncontact ACL rupture particularly in women and in young athletes [11,21,22,40]. From our results, this relationship can be extended to a large population whatever gender or age when this parameter is analyzed alone. However, in multivariate model, this parameter was not powerful enough to be taken into consideration to improve the diagnosis accuracy of noncontact ACL injury.

From our results, non-contact ACL injury prevention could be proposed by hamstring strengthening, particularly in a population under the age of 23.5 years, or in case of knee laxity ≥ 4.5 mm. An increased of the relative hamstring co-contraction with the quadriceps may lead to an increased knee flexion, a reduced knee abduction and a reduced anterior tibial shear during dynamic motion [29]. In addition, hamstring knee strength should control knee rotation when a dynamic knee valgus is combined in closed kinetic chain to avoid ACL impingement [37].

One limitation of the present study was to use a method focusing on many intrinsic factors, without studying all factors such as knee geometrical morphology using MRI while many results had been published on this subject [20]. This choice was made so as to study factors easy to measure in clinical practice in order to propose a “predictive approach” of ACL injury without expensive medical means. In addition, MRI measurements have the limit of not performing the knee in support, which may explain some controversies [35]. However, radiological or posturometric examinations could be of greater value. A second limitation was to consider that both limbs of a patient were symmetrical before injury. Indeed, we cannot exclude that some patients might have differences between the injured limb and the non-injured one. The ACL injury risk factors of the non-injured limb may be not exactly the same as those of the injured limb [38,39]. Nevertheless, our cross-sectional method made it possible to be certain of ACL injury of one of the two knees considered identical. At last, the studied population was an athlete population who practice sports involving knee injury risks. The conclusions of this work are therefore probably not applicable to all populations, especially to non-sports populations.

## 5. Conclusions

Non-contact ACL injury was associated with age, passive knee extension and weak hamstring knee strength in an ACL reconstruction population whatever gender. Passive knee valgus is strongly associated with the female population. Unfortunately, all these factors are not changeable. Only hamstring isokinetic strength could be improved by strengthening. These modifiable intrinsic factors are also associated with different subpopulations particularly in men, but also in young athletes under 23.5 years old and in populations with an anteroposterior knee laxity upper 4.5 mm. According to these results, hamstring strengthening could be achieved especially in these populations. However, this preventive attitude needs to be confirmed by prospective comparative studies in future.

## Figures and Tables

**Table 1:** Sport participation and mechanisms of ACL injury before ACL reconstruction.

Sports	Noncontact Group (*n* = 195)	Contact Group (*n* = 112)
Soccer, *n* (%)	79 (40.5%)	56 (50%)
Basketball, *n* (%)	38 (19.5%)	12 (10.7%)
Ski, *n* (%)	29 (14.9%)	8 (7.1%)
Handball, *n* (%)	16 (8.2%)	6 (5.4%)
Rugby, *n* (%)	5 (2.6%)	7 (6.3%)
Other sports, *n* (%)	23 (20.5%)	28 (14.4%)

**Table 2:** Comparison of associated factors according to the mechanism of ACL injury in all population (Univariate analysis).

	Non Contact Group (*n* = 195)	Contact Group (*n* = 112)	OR	95%CI	*p*
Gender male (*n* = 206)	61.2%	38.8%	0.73	0.44–1.20	0.22
Gender female (*n* = 101)	70.7%	29.3%	1.50	0.87–2.58	0.17
Age (years)	27 ± 9	24 ± 8	1.04	1.01–1.07	0.002
Weight (kg)	72 ± 13	69 ± 10	1.02	1.00–1.04	0.04
Height (cm)	174 ± 8	173 ± 8	1.01	0.98–1.04	0.41
BMI (kg/m^2^)	23.7 ± 3.6	22.9 ± 2.5	1.08	1.00–1.17	0.04
Q60 (Nm/kg)	2.49 ± 0.50	2.64 ± 0.45	0.53	0.33–0.87	0.01
Q180 (Nm/kg)	1.60 ± 0.31	1.71 ± 0.31	0.34	0.16–0.73	0.006
H60 (Nm/kg)	1.30 ± 0.29	1.42 ± 0.29	0.25	0.11–0.56	0.001
H180 (Nm/kg)	0.98 ± 0.22	1.07 ± 0.22	0.17	0.06–0.49	0.001
H/Q60 (%)	52.5 ± 8.1	54.1 ± 8.2	0.09	0.006–1.70	0.11
H/Q180 (%)	61.6 ± 10.5	63.1 ± 9.6	0.23	0.02–2.3	0.21
P K VL (mm)	1.8 ± 2.8	0.7 ± 1.7	1.24	1.09–1.40	0.001
P K E (°)	6.2 ± 4.4	4.0 ± 4.1	1.13	1.06–1.19	0.001
Knee Laxity (mm)	3.8 ± 1.6	3.3 ± 1.6	1.19	1.03–1.37	0.01

Abbreviations: BMI: Body Mass Index; Q60: Isokinetic quadriceps strength at 60°/s; H/Q: Hamstring-to-Quadriceps ratio; P K VL: Passive Knee Valgus; P K E: Passive Knee Extension; OR: Odd Ratio; 95%CI: Confidence Interval at 95%.

**Table 3:** Multivariate models of noncontact ACL injury in all population and after gender or cut-offs variables selection (gender; age ≤ 23.5 year; BMI ≥ 22.5 kg/m^2^ and Knee Laxity ≥ 4.5mm).

	*B*	Wald	OR	95%CI	*p*
All population					
Age	0.049	10.0	1.05	1.02–1.08	0.001
H strength at 180°/s	−1.30	5.4	0.27	0.09–0.80	0.01
P K E	0.135	19.1	1.14	1.07–1.21	0.001
Constant	−0.055	0.005	0.15		
Men (*n* = 206)					
Age	0.054	7.76	1.01	1.01–1.09	0.005
H strength at 180°/s	−1.56	3.89	0.04	0.04–0.98	0.048
P K E	0.136	14.0	1.06	1.06–1.23	0.001
Constant	0.065	0.003	1.06		
Women (*n* = 101)					
P K VL	0.244	6.66	1.27	1.06–1.53	0.01
Constant	0.238	0.72	1.18		
Age ≤ 23.5 year (*n* = 145)					
P K E	0.088	4.37	1.09	1.01–1.18	0.03
H strength at 60°/s	−1.26	4.74	0.28	0.09–0.88	0.02
Constant	1.55	3.00	4.75		
BMI ≥ 22.5 kg/m^2^ (*n* = 180)					
P K VL	0.193	5.89	1.21	1.03–1.41	0.01
P K E	0.152	11.4	1.16	1.06–1.27	0.001
Age	0.056	8.26	1.05	1.01–1.09	0.004
Constant	−1.98	9.1	0.13		
KT1000 ≥ 4.5 mm (n = 109)					
H strength at 180°/s	−2.51	5.93	0.08	0.01–0.61	0.01
Constant	3.46	10.4	31		

Abbreviations: OR: Odd Ratio; 95%CI: Confidence Interval at 95%. H: Hamstring; P K E: Passive Knee Extension; P K VL: Passive Knee Valgus.

**Table 4:** Cut-offs of associated factors with noncontact ACL rupture identified by ROC curve area and Youden index.

	ROC Curve Area	95%CI	Se (%)	Sp (%)	LR+	LR−
P K E = 4 degrees	0.643	0.579–0.708	61	58.9	1.48	0.66
P K VL = 15 mm	0.605	0.542–0.669	41	79.5	2	0.74
Age = 23.5 years	0.602	0.538–0.667	67.2	47.3	1.27	0.69
BMI = 22.5 Kg/m^2^	0.556	0.491–0.621	60	46.4	1.12	0.86
Knee Laxity = 4.5 mm	0.585	0.519–0.650	40.5	73.2	1.51	0.81

Abbreviations: ROC: Receiver Operating Characteristics; 95%CI: 95% confident interval; Se: Sensitivity; Sp: Specificity; LR+: positive likelihood ratio; LR−: negative likelihood ratio.

## Data Availability

The data presented in this study are available on request from the corresponding author. The data are not publicly available due to ethical reasons.
